# Environmental Contaminants in Fish Products: Food Safety Issues and Remediation Strategies

**DOI:** 10.3390/foods13213511

**Published:** 2024-11-02

**Authors:** Pierina Visciano

**Affiliations:** Department of Bioscience and Technology for Food, Agriculture and Environment, University of Teramo, Via R. Balzarini 1, 64100 Teramo, Italy; pvisciano@unite.it; Tel.: +39-0861266911

**Keywords:** fish, water products, persistent organic pollutants, metals, microplastics, remediation

## Abstract

The intentional or accidental presence of environmental contaminants, such as persistent organic pollutants, metals, and microplastics, can harm the aquatic ecosystem and their living organisms, as well as consumers of seafood. This study provides an overview of marine pollution caused by various chemicals and their toxicity to both the environment and humans. In addition to regulatory limits established for some contaminants, monitoring and management policies should mandate activities such as bioremediation and the use of carbon-based composite photocatalysts to reduce or eliminate these compounds.

## 1. Introduction

Fish products are considered healthy and high-quality foods as they are sources of numerous nutrients; however, the potential presence of harmful chemicals can have adverse effects on human health [1]. Environmental contaminants can be present in the marine ecosystem, where they remain for many years and are therefore better known as persistent organic pollutants (POPs). They include polycyclic aromatic hydrocarbons (PAHs), dioxins, polychlorinated biphenyl (PCBs), and flame retardants such as polybrominated diphenyl ethers (PBDEs) [2]. Many POPs are hydrophobic compounds and show a high affinity for lipid-rich tissues [3]. They are resistant to photolysis and biodegradation, and this promotes their retention in water, sediments, and marine organisms [4]. Additional contaminants that can compromise the safety of fish products include some metals such as lead (Pb), cadmium (Cd), mercury (Hg), and arsenic (As), which can also be found in high concentrations [5].

According to Commission Regulation (EU) 915/2023 [6], fish and fishery products that contain contaminants at levels exceeding the maximum limits cannot be placed on the market and/or used as a raw material or as an ingredient in processed products [7]. The environmental contaminants with regulatory limits (Table 1) include metals, dioxins, PCBs, PAHs, and perfluoroalkyl substances (PFASs). The sum of dioxins and dioxin-like PCBs is related to polychlorinated dibenzo-para-dioxins (PCDDs) and polychlorinated dibenzofurans (PCDFs), while the sum of non-dioxin-like PCBs consists of PCB28, PCB52, PCB101, PCB138, PCB153, and PCB180 congeners. The sum of PAHs refers to benzo(a)pyrene, benzo(a)anthracene, benzo(b)fluoranthene, and chrysene, while PFASs are classified as single or sum of perfluorooctane sulfonic acid (PFOS), perfluorooctanoic acid (PFOA), perfluorononanoic acid (PFNA), and perfluorohexane sulfonic acid (PFHxS). The abbreviations of the environmental contaminants investigated in this study are reported in Table 2.

The maximum levels differ in fish species. The limit of Cd is 0.050 mg/kg in most of them, but it increases up to 0.10 mg/kg in mackerel, tuna, and bichique, and 0.15 and 0.25 mg/kg in bullet tuna and anchovy, sardine, and swordfish, respectively. Similarly, the limits for Hg vary between 0.5 and 1 mg/kg due to biomagnification in large fishes such as tuna, shark, and swordfish, while it is lower (0.30 mg/kg) in small-sized fishes. The sum of non-dioxin-like PCBs is higher in wild-caught fish, i.e., 200 and 300 ng/g in spiny dogfish and eel, respectively. The maximum levels for the sum of PFASs are 8 and 45 μg/kg in Baltic herring, bonito, burbot, and European sprat and some benthonic or wild fish species, respectively. The complete list of species for each group of contaminants is reported in the Annex of Commission Regulation (EU) 915/2023.

The limits for benzo(a)pyrene (BaP) and the sum of PAHs are currently established only for fresh, chilled, and frozen bivalve mollusks, although in the previous regulations, they were considered also for unprocessed fish, crustaceans, and cephalopods; however, their maintenance was demonstrated to be no longer appropriate, as they are quickly metabolized (Commission Regulation EU 835/20122) [8]. Today, they must be monitored in smoked fish products as well as smoked bivalve mollusks at maximum concentrations of 2.0 and 12 μg/kg, and 6.0 and 35 μg/kg for BaP and the sum of PAHs, respectively. Additionally, fish products that have undergone heat treatment (i.e., grilling and barbecuing), potentially resulting in PAH formation, must be compliant with maximum levels of 5.0 and 30 μg/kg for Bap and the sum of PAHs, respectively.

**Figure 1:**
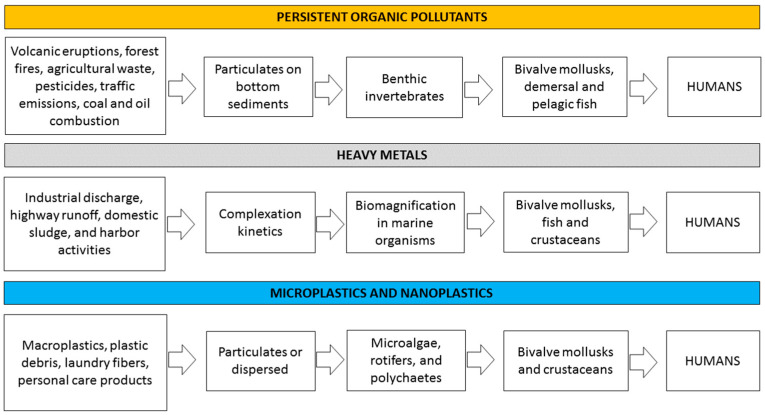
Pathways of movement of environmental contaminants in the marine food web.

Environmental contaminants are urgent issues for both human health and the marine ecosystem. They represent a global concern due to their persistence in the environment, the ability to biomagnify and bio-accumulate in fish products that reach humans through the food web, and their adverse effects on marine organisms, human beings, and the environment [9]. They can be grouped into unintentionally and intentionally produced chemicals that are detected worldwide, highlighting their ubiquity in water, sediment, and living organisms [10]. In recent decades, emerging threats have developed into a growing global problem due to the degradation and fragmentation of plastic debris into smaller particles, i.e., microplastics (MPs) and/or nanoplastics (NPs), which can be ingested by marine biota through passive water filtration or feeding [11]. The pathways of movement of the investigated categories of environmental contaminants into the marine food web are shown in Figure 1.

**Figure 2:**
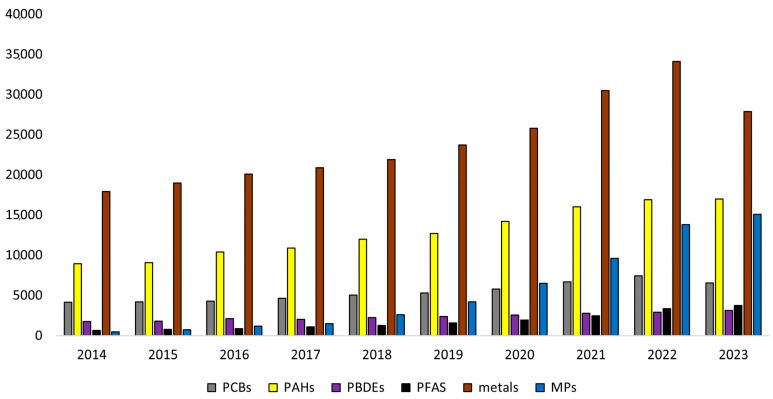
Number of publications per year (2014 to 2023) in Google Scholar regarding the main groups of environmental contaminants investigated in this study.

**Figure 3:**
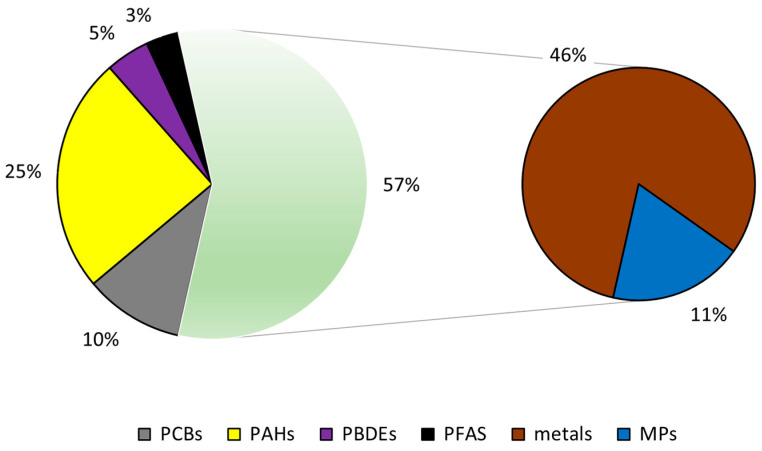
Proportions of environmental contaminants investigated in this study calculated as averages of topics of publications (2014 to 2023) in Google Scholar.

A literature search in Google Scholar using the various investigated groups of contaminants in marine ecosystems as text words showed an increasing trend during the last 10 years (2014 to 2023), which underlines the particular concern linked to this topic (Figure 2). The proportions of the different groups were calculated as the average of the publications from 2014 to 2023 (Figure 3). The percentage related to metals and MPs corresponded to 57%; the remaining 43% consisted of distinct groups of POPs. The number of publications on metals and MPs showed an increase, while those referring to POPs were more stable in the years analyzed. Metals are considered a severe environmental threat due to their long persistence and non-degradability, although their environmental behavior and toxicity are strongly dependent on their chemical forms. Metal speciation (i.e., the selective extraction of metals into different physicochemical fractions) is now becoming essential for a more realistic estimate of the environmental impact of a particular element since it offers useful information on its chemical nature or bioavailability, which can influence the metal content in marine organisms [12]. Regarding MP studies, the scientific community is currently engaged in a critical discussion on their heterogeneity in terms of chemical composition, size, and form, as well as their biodistribution in living organisms [13]. Although several studies have demonstrated the presence of environmental contaminants in the marine ecosystem, and consequently in fish products, complete knowledge of strategies for their limitation and removal is lacking. The current review focuses on known and emerging environmental contaminants and their toxicity to marine organisms, humans, and the environment, and aims to fill these knowledge gaps by indicating future perspectives.

## 2. Persistent Organic Pollutants

### 2.1. Polychlorinated Biphenyls

These compounds are ubiquitous and bio-accumulative pollutants that easily reach the human food chain. PCBs are mainly of industrial origin, as they are used as insulators and dielectric fluids in transformers and capacitors, flame retardants, oil additives, and lubricants [14]. The chlorination of the biphenyl ring generates 209 congeners that differ in toxicity and bioavailability depending on the number and position of chlorine atoms [15]. Conversely, PCDDs and PCDFs have never been produced intentionally, but they are released into the environment during natural combustion processes (forest fires and volcanic eruptions) and/or as by-products of various manufacturing activities (paper whitening; production of pesticides, herbicides, and fungicides; iron smelting; and cement kilning) [16].

PCDDs and PCDFs include 75 and 135 congeners, respectively, and they are called dioxin-like PCBs since they exhibit mechanisms of toxic action similar to dioxin. Their adverse effects differ considerably, and only some congeners show significant toxicity because of the replacement of hydrogen with chlorine atoms, at least in the 2, 3, 7, and 8 positions [2]. The 2,3,7,8-tetrachlorodibenzo-para-dioxin (2,3,7,8-TCDD) has been classified as a human carcinogen (group 1) by the International Agency for Research on Cancer (IARC), causing lung cancer, soft tissue sarcoma, non-Hodgkin lymphoma, breast cancer, rectal cancer, and myeloid leukemia [17]. Dioxins are also responsible for skin lesions (chloracne), immune impairment, and neurological disorders [18]. Human exposure to all dioxin-like compounds is usually designed in terms of the toxic equivalence quotient (TEQ), which is calculated by standardizing the individual congener levels detected in each sample, multiplying them with the appropriate toxic equivalency factor (TEF), and summing these normalized values. TEFs have been established by the World Health Organization and are calculated relative to 2,3,7,8-TCDD [17].

In the aquatic environment, PCBs, PCDDs, and PCDFs bind to suspended particulates, forming a reservoir on bottom sediments, and are taken up by benthic organisms, which can reach the highest levels of the marine food chain if ingested by fish and the latter by humans [19]. Because of their hydrophobic properties, they are likely to accumulate in oily fish species [20,21], but other factors such as the contamination of fishing areas can also influence dietary exposure to these compounds [22]. Bivalve mollusks filter nutrients from seawater and can absorb dioxin-like and non-dioxin-like PCBs, but they are unable to metabolize and/or excrete these compounds, and therefore, they are further sources for humans [23]. In particular, mussels and oysters have been considered bioindicators of marine contamination, and in the last decades, many coastal monitoring programs have been established, using mussels as sentinel [24]. Visciano et al., (2015) [25] reported total non-dioxin-like PCBs concentrations ranging from 1.96 to 2.43 ng/g wet weight (ww) in samples of *Chamelea gallina* collected from different sampling sites along the coast of the Central Adriatic Sea, whereas dioxin-like PCBs were detected at levels of between 0.103 and 0.116 pg/g ww. The sum of WHO-TEQs for PCDDs and PCDFs ranged from 0.027 to 0.049 pg/g ww.

### 2.2. Polycyclic Aromatic Hydrocarbons

PAHs are generated via incomplete combustion of natural (i.e., forest fires, volcanic eruptions) and/or anthropogenic sources such as fossil fuels, petroleum refining, coal gasification, and waste incineration [26]. They are made of two or more fused benzene aromatic rings with different structural configurations, and the higher their molecular weight, the lower their aqueous solubility [27]. In marine environments, PAHs tend to remain adsorbed on particulate matter for long periods due to their high hydrophobicity and can accumulate in the fatty tissues of fish after ingestion or sorption through skin or gills [28]. Paik et al., (2024) [29] reported the presence of PAHs in various marine products, particularly shellfish, which showed the highest detection rate (70%), followed by fish (19%) and crustaceans (8%), in which chrysene was the most prominent PAH congener. These compounds can also be produced during some cooking processes, such as smoking, due to the incomplete combustion of wood used for smoke generation [30], or charcoal grilling, because of direct contact of the food with an open flame [31]. Gao et al., (2022) [32] demonstrated that this process increased the PAH content in charcoal-grilled fish samples, with phenanthrene, fluoranthene, and pyrene as the main PAHs detected, while in raw fish, naphthalene was found to be the dominant compound.

Among the potential sources of PAHs for humans, dietary intake is the main route for individuals who are non-smokers and/or non-occupationally exposed [33]. Seven PAHs, i.e., benzo(a)pyrene, chrysene, benzo(a)anthracene, dibenzo(ah)anthracene, benzo(k)fluoranthene, benzo(b)fluoranthene, and indeno(1,2,3-cd)pyrene, are classified as carcinogenic to humans [17]. Carcinogenicity is associated with their property of getting attached to DNA-forming DNA adducts, which are responsible for generating several disorders that ultimately result in tumor development [34]. The United States Environmental Protection Agency (USEPA) listed 16 priority PAHs out of a total of approximately 2,000 PAH compounds, including naphthalene, acenaphthylene, acenaphthene, fluorene, phenanthrene, anthracene, fluoranthene, pyrene, benzo(a)anthracene, chrysene, benzo(a)pyrene, benzo(b)fluoranthene, benzo(k)fluoranthene, indeno(1,2,3-c,d)pyrene, dibenzo(a,h)anthracene, and benzo(g,h,i)perylene due to their genotoxic, mutagenic, and carcinogenic properties. PAH metabolites that are bio-transformed by cytochrome P450 can bind DNA and result in mutagenicity or carcinogenicity [35].

### 2.3. Perfluoroalkyl Substances

PFASs are a class of synthetic organic compounds divided into two main groups: perfluoroalkyl carboxylic acids and perfluoroalkyl sulfonic acids, and PFOA and PFOS are typical representatives of these two classes, respectively [36]. They are produced or used to manufacture other products such as coatings, paints and varnishes, fire-fighting foams, food packaging materials, plastic, rubber and resins, and personal care products [37]. The main human exposure routes are dietary ingestion (particularly drinking water), inhalation of air and dust particles, hand-to-mouth contact, and dermal absorption [38]. These compounds have various effects on human health, including thyroid disease; increased cholesterol levels; liver, lung, kidney, or testicular cancers; oxidative stress; and apoptosis. In specific population groups, such as pregnant women and infants, they can interfere with natural hormones, reducing the chances of pregnancy or causing elevated blood pressure with potential pre-eclampsia and decreasing birth weight and causing growth, learning, and behavioral issues, respectively [39].

Wastewater treatment plants are among the main sources of PFASs in surface water. Benthic invertebrates in the marine ecosystem are often exposed to PFASs through the ingestion of sediment particles and therefore contribute to the diet of fish entering the marine food web [40]. As many PFASs bioaccumulate and biomagnify in aquatic environments, fish can become an important source of these contaminants for consumers [41]. Fair et al., (2019) [42] reported mean PFAS levels ranging from 12.7 to 33.0 ng/g wet weight in whole fish of different species collected from South Carolina, and 6.2–12.7 ng/g ww in fillets. PFOS was the most abundant compound in each examined fish species, comprising 25.5–69.6% of the total PFASs. Similarly, PFOS was predominant in fish and crustacean samples from the Belgian North Sea, at mean concentrations of 24 and 5.6 ng/g ww, respectively [43]. A significant relationship between PFAS concentrations and various ecological characteristics (species, body size, habitat, feeding guild, and location) was observed in 18 different marine species coming from a typical North Atlantic Ocean food web. The benthic omnivores and pelagic piscivores showed the highest mean PFAS concentrations up to a maximum of 10.5 and 8.50 ng/g ww, respectively [44]. Zafeiraki et al., (2019) [45] examined wild and farmed aquatic animals collected in the Netherlands and found maximum levels for the sum of PFOA, PFNA, PFHxS, and PFOS of 9.4 and 3.2 ng/g ww in seabass and cod from the North Sea, respectively, while in farmed fish, they were below 1.3 ng/g ww, as reported in other studies [46]. PFOS was generally detected at higher levels and higher frequency than other PFASs [45].

### 2.4. Flame Retardants and Other POPs

Flame retardants are chemical compounds that are applied to materials to prevent or slow the growth of fire by interfering with one or several stages of the process of fire. In general, they are divided based on whether they contain bromine, chlorine, phosphorus, nitrogen, metals, or boron. The main groups include halogenated, organophosphorus, and aliphatic or aromatic brominated flame retardants [47]. PBDEs are a class of brominated aromatic compounds with a basic structure consisting of two phenyl rings linked by an ether bond. They have widespread use in the construction of materials, furniture, and electric and electronic equipment. The following ten congeners are particularly studied: BDE-28, -47, -49, -99, -100, -138, -153, -154, -183, and -209. The predominant congeners found in fish are the result of debromination of BDE-209, -99, or -183 into lower brominated congeners such as BDE-47 [48]. Total PBDE concentrations ranging from 1.69 to 47.6 ng/g lipid weight (corresponding to 0.01–0.20 ng/g wet weight) were detected in edible fish species from the wide-open South China Sea. BDE-47, -209, -100, and -154 were the dominant target PBDE congeners, representing 49.2, 17.2, 9.93, and 7.43%, respectively [49]. An investigation on PBDE levels in freshwater fish from rivers in southeastern Virginia (USA) demonstrated a decrease of >75% over 20 years, even though they were still detected in 93% of the samples at a maximum concentration of 16,300 ng/g lipid weight. BDE-47 was the dominant PBDE congener (93%), followed by BDE-99 (67%) [50]. PBDE-28, -47, -99, -156, and -209 were found in all liver samples of marine fish (Atlantic cod and turbot) as well as in blood samples of consumers from two rural Newfoundland communities [51]. Several studies reported that exposure to PBDEs is associated with an increased risk of thyroid disease, diabetes, and metabolic syndrome, as well as cancer and mortality [52]. The transfer of PBDEs from maternal blood to the placenta and human milk has also been reported [2].

Additional POPs include organochlorine pesticides (OCPs), polychlorinated naphthalene (PCN), dechlorane plus (DP), and hexachlorobenzene (HCB). These pollutants tend to biomagnify at high concentrations and accumulate in the living organisms present at the highest trophic level of the aquatic food chain. Severe health effects may involve neurobehavioral impairments, congenital disabilities, immunodeficiency disorders, and hormonal imbalances [53]. Short-term exposure to OCPs can cause euphoria, perceptual disturbances, seizures, agitation, or lethargy [54], while anorexia, hepato- or renal toxicity, neurological disturbances, and skin irritation are associated with long-term exposure [55]. PCN causes thymus atrophy and hematological or endocrine disturbances [56]. Neurobehavioral anomalies and endocrine disruption effects are linked to DP contact [57], while HCB is responsible for porphyria, thyroid dysfunctions, and immunological, neurological, and reproductive disorders [58].

**Figure 4:**
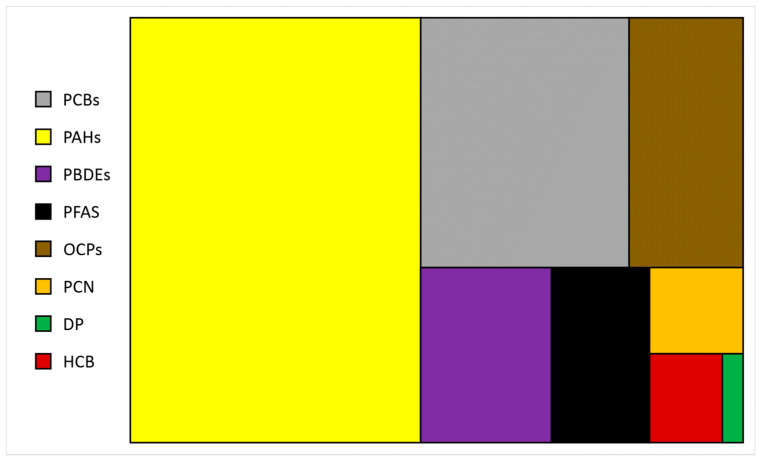
Proportions of persistent organic pollutants in marine ecosystems calculated as averages of topics of publications (2014 to 2023) in Google Scholar.

The average distribution of publications in Google Scholar regarding the various POPs investigated in the last 10 years (Figure 4) showed that the majority of the studies focused on PAHs (47%), followed by PCBs (20%), OCPs (11%), and PBDEs (9%). These are a wide variety of anthropogenic compounds of organic nature that are used for several purposes and reach the marine environment through different pathways. While the main sources of PAHs and PCBs are industrial activities, combustion processes, motor vehicles, incinerators, etc., OCPs are derived from the intensification of agricultural practices [59].

## 3. Metals

In the marine environment, metals can remain in solution or in suspension and precipitate to the bottom, or they can be absorbed by aquatic organisms and get biomagnified through the food chain up to humans [60]. Benthic fish residing near sediments feed primarily on organic debris and accumulate metals in their tissues depending on some intrinsic factors such as enzymes and intestinal pH [61]. If these contaminants are not broken down in the organisms, their concentrations tend to rise as they move up the aquatic food chain. When there is a transfer of contaminants between trophic levels, a coefficient greater than 1 indicates biomagnification, whereas values less than 1 show that the contaminants are not biomagnified across the organisms [62]. Some authors reported trophic magnification factors (TMFs) of 1.29, 0.96, and 0.11 for total Hg, Pb, and Cd, respectively, showing a positive trophic magnification slope only for total Hg in marine waters [63]. Yu et al., (2022) [64] calculated TMFs for methylmercury (MeHg) in different marine organisms (fish, crustaceans, and mollusks) based on diet (zooplankton or phytoplankton) and found values of 1.26, 1.43, and 1.42 and 1.29, 1.44, and 1.42 for crustaceans, fish, and mollusks, respectively.

Apart from natural sources (weathering, atmospheric precipitation, and volcano eruptions), metals arise in the environment from a variety of anthropogenic activities, such as the release of untreated effluents from various industries, leaching from agricultural practices, and production of microelectronic equipment, paints, plastics, batteries, and medical devices [65]. Humans are particularly exposed to metals through diet. MeHg is mainly present in fish and other seafood [66], while Pb and Cd can occur in many other food categories of terrestrial origin, such as fruits and vegetables, mushrooms, and spices, as well as foods obtained from animals (meat and dairy products). Metals induce both carcinogenic and non-carcinogenic effects (mainly neuronal and endocrine), with a higher risk in children than adults [67].

Many studies have suggested a relationship between exposure to the metal mixture of Pb, Cd, Hg, and As and neurodevelopmental diseases, including autism spectrum disorders and attention deficit and hyperactivity disorders [68]. It has been reported that Pb exposure during the development period can cause cognitive impairment and inattention in children and inhibit hippocampal synaptic transmission in mice. The hippocampus, belonging to the limbic system, encodes learning and memory, stress, and anxiety regulation [69]. The neurologic damage caused by Pb is permanent. Its toxicity is due to the faculty to replace calcium in biological processes interfering with calcium ion flow. This metal can remain for many years in bone tissue, where it integrates into hydroxyapatite and interferes with cell signaling, maturation, and differentiation, leading to impaired fracture healing and increased risk of osteoporosis [70]. Chen et al., (2024) [71] demonstrated that As and Pb co-exposure significantly activated proteins associated with carcinogenesis in brain organoids using optic vesicles as experimental models for understanding neurological-related diseases. Inorganic As shows higher toxicity than its methylated forms. It is classified as carcinogenic to humans (group 1) by IARC [72] and can increase the risk of cancer in the lungs, liver, skin, bladder, and kidneys [73]. Furthermore, it accumulates in the skin, causing arsenical keratosis and hyperpigmentation [74].

Neurological, pulmonary, and renal effects have been observed in humans exposed to the three forms of Hg, i.e., organic Hg, such as MeHg and dimethyl Hg (DMeHg), or inorganic Hg, including elemental Hg (Hg0) and oxidized Hg (HgII) [64]. Acute exposure to high levels of all Hg forms can be fatal, while a chronic introduction of MeHg through diet is associated with adverse neurodevelopmental outcomes as well as cardiovascular risks. Some population groups, such as pregnant women and newborns, are more vulnerable to MeHg as it can cross both the placenta and the blood–brain barrier of the fetus, leading to motor and/or cognitive deficits [75,76].

A positive correlation between Cd exposure and breast, bladder, and colorectal cancers has been shown through toxic mechanisms such as oxidative stress, apoptosis, autophagy, and DNA damage [77]. Cd is classified by the IARC as carcinogenic to humans (group 1) [72] and can accumulate in the human body for many years (half-life of 25–30 years) [78]. Metals are nonbiodegradable and bioaccumulate in different parts of the human body, e.g., lead is deposited in bone tissue, cadmium in the kidneys, and mercury has a particular trophism for the nervous system. Nevertheless, living organisms may detoxify metal ions by binding to proteins or depositing them in intracellular granules to be excreted via feces [79]. Selenium is an essential micronutrient in animals and humans, with antioxidative properties as well as important roles in the regulation of thyroid hormone metabolism; however, it can be toxic at high concentrations [66]. Tributyltin oxide belongs to the organometallic family of tin compounds used as biocides, disinfectants, and antifoulants; its effects have been observed only in animals, including thymus atrophy, depletion of T-lymphocytes in the spleen and lymph nodes, and decreased leukocytes and hemoglobin mass [80]. Tin compounds are a global threat to marine ecosystems, where they remain stable for many years; they withstand lengthy periods of time in sediment and are easily accumulated in benthic organisms; and they have toxic effects on aquatic populations, even at very low doses. Tributyltin has been investigated more than other tin components in seafood, with an overall average estimate of 182.33 ng/g [81]. Shu et al., (2023) [82] detected orgatin compounds at concentrations ranging from 13 to 2,900 ng Sn/g dry weight (dw) in mollusks (mean 200 ng Sn/g dw), and from 0.81 to 960 and 3.9 to 420 ng Sn/g dw in crustaceans and fish, respectively.

## 4. Microplastics and Nanoplastics

MPs are plastic particles ranging from 1 μm to 5 mm, with a primary or secondary origin. Primary MPs are derived from personal care products like liquid soap, exfoliating scrubbers, and cleaning supplies or pellets for the production of polymers, while secondary MPs originate from the fragmentation of larger plastic items or abrasion of synthetic textiles, car tires, and paint flakes [83,84]. The size distinction with NPs considers the latter as smaller than 1 μm [85] or 100 nm [86]. It has been demonstrated that the bioactivity of plastic particles is strictly size-dependent, and NPs exhibit the greatest invasiveness and capacity to penetrate cellular membranes across various organs such as the lungs and excretory organs, or the heart and brain [87]. The interaction between NPs of sizes 0.25 and 1 μm and human colorectal cancer cell lines could enhance cell migration and potentially promote metastasis [88].

**Figure 5:**
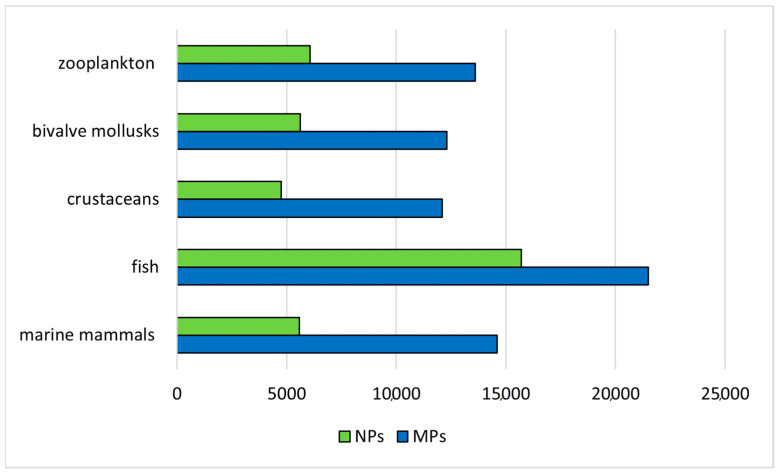
Numbers of publications in Google Scholar on the contamination of aquatic products by microplastics and nanoplastics in the last 10 years (2014–2023).

Humans are exposed to MPs through inhalation, ingestion, and dermal contact, potentially leading to chronic inflammatory alterations [89]. Plastic additives (i.e., antioxidants, biocides, flame retardants, heat stabilizers, plasticizers) incorporated into MPs to improve the properties of the polymers have been found to cause adverse biological effects at relatively low concentrations [90]. Additionally, MPs can sorb and accumulate both organic and inorganic contaminants such as PAHs, PCBs, PBDEs, toxic metals, and pharmaceutical compounds, which can be released into marine organisms ingesting them, and reach humans through the food web. The sorption of chemicals is influenced by some plastic properties, such as surface charge, surface area, molecular chain arrangement, functional groups present, and the acid–base character [91]. Most POPs are hydrophobic and insoluble in water, so they tend to accumulate in sediment and biota. As MPs have a large surface area compared to their volume, the attachment of these pollutants, especially PAHs, PCBs, and OCPs, is particularly favored due to the hydrophobic interactions [92]. Heavy metals can bind to MPs through various mechanisms such as adsorption, ion exchange, and chelation. The process requires the interchanging of ions between the surface of MPs and the surrounding environment [93]. Various types of MPs such as polystyrene (PS), polypropylene (PP), polyethylene (PE), polycarbonate (PC), polyethylene terephthalate (PET), and polyvinyl chloride (PVC) have been found to be associated with POPs in different water bodies [94]. Some studies have demonstrated that PE showed higher sorption capacities for hydrophobic organic compounds than PVC and PS particles [95,96]. Bai et al., (2024) [97] reported that MPs significantly increased Hg accumulation, affecting both the development and reproduction of marine copepods, while Rial et al., (2023) [98] demonstrated that the desorption and subsequent dermal uptake by mussels and sea urchin embryos of Hg, chlorpyrifos, and fluoranthene through MPs acting as vectors was the main origin of their toxicity. Apart from chemicals, MPs can also transport microorganisms, which are ingested and delivered to the tissues, protected from the immune system of the body [99]. Alterations to the gut microbiome may lead to adverse effects, such as the proliferation of harmful species, an increase in intestinal permeability, and endotoxemia [89,100]. The numbers of publications in Google Scholar referring to the contamination of marine organisms by MPs and NPs in the last 10 years (2014–2023) are shown in Figure 5.

MPs can be present in soils, marine water, sediments, the atmosphere, and the human body. Multiple kinds of MPs have been found in human blood [101], feces [102], and placenta [103]. Human exposure through the ingestion of foods such as seafood, terrestrial animal meat, and plant-based products obtained with different technologies (unprocessed and minimally- and highly processed) has recently been investigated. The average ± standard deviation of MP content was 0.3 ± 0.7 MP/g across all products, ranging from mean particle concentrations of 0.01 ± 0.01 MP/g in chicken breasts and pork loin chops, to 1.3 ± 1.9 MP/g in breaded shrimp. The mean number of MPs consumed in a single serving of seafood, terrestrial meat, and plant-based protein was 120 ± 320, 32 ± 61, and 40 ± 69 MPs, respectively. Highly processed products had more MP particles than minimally processed foods, perhaps because they were subjected to contact with plastic food production equipment (e.g., conveyor belts and workers’ clothing) for greater amounts of time. Plastic fibers were the predominant morphology found, followed by fragments and rubber [104].

## 5. Strategies for Environmental Contaminant Restriction and Removal

### 5.1. Regulatory Efforts

The Stockholm Convention on POPs was adopted on 22 May 2001 and entered into force on 17 May 2004, but ordinary meetings of the Conference of the Parties bound by this Convention are held at regular intervals to develop guidance regarding the best environmental practices to protect human health and the environment from POPs [105], including measures to reduce or eliminate the release of POPs in the environment from both intentional and unintentional production. The Secretary-General of the United Nations acts as the Depository of this Convention. The Annexes to the Stockholm Convention comprise substances that must be eliminated and/or whose production must be prohibited (Annex A), substances whose production and use must be restricted (Annex B), and substances for which only measures to reduce the total release from anthropogenic sources must be taken (Annex C) because they are unintentionally formed and released into the environment. Several OCPs, PFASs, and PBDEs are included in Annex A of the revised version of the Stockholm Convention, while PFOS is reported in Annex B as an intermediate in the production of chemicals to be used solely in insect baits for agricultural use. The use of PCBs in equipment such as transformers, capacitors, or other receptacles containing liquid stocks must be unauthorized by 2025. HCB, PCDDs, and PCDFs are reported in Annex C [106].

The United States (US) has taken a leading role in reducing and/or eliminating POPs and their release on a regional and/or global basis. A legally binding regional protocol was signed with other nations (including European countries, Canada, and Russia) under the Convention on Long-Range Transboundary Air Pollution. Furthermore, the US, Canada, and Mexico established the Commission for Environmental Cooperation under the North American Agreement on Environmental Cooperation, which in turn developed a regional initiative on the management of these chemical contaminants through Regional Action Plans, which identify activities that reduce or eliminate risks [9]. USEPA has established two lists of contaminants to be monitored in fish and shellfish, as they have been found to occur at concentrations that may be of concern for human health. The first is a list of contaminants for which USEPA demonstrated oral toxicity in humans, issuing advisory programs with consumption limits. They include PFASs, PAHs, dioxins, furans and PCBs, organochlorine and organophosphate pesticides, flame retardants (BDE-47), and metals (lead, cadmium, inorganic arsenic, methylmercury, selenium, and tributyltin) [107]. USEPA also developed a series of fish consumption advisories that provide guidance only and do not constitute a regulatory requirement. The risk-based consumption limits (fish meals/month) for noncarcinogenic health endpoint related to metal concentrations in fish tissues are reported in Table 3, while Table 4 shows the risk-based consumption limits for carcinogenic health endpoint related to PAH, PCB, and dioxin/furan levels [80]. Some sensitive consumer groups are at higher risk than the general population, such as sports and subsistence fishers eating higher-than-average quantities of fish and pregnant women and children because of their proportionally higher consumption rates and/or increased susceptibility to adverse toxicological effects [75].

Also, the European Union has made efforts to handle environmental pollution by enacting some stringent rules. Regulation (EU) 1021/2019 of 20 June 2019 [108] aims to protect human health and the environment from POPs by prohibiting or restricting the manufacturing, placing on the market, and use of substances listed in the Stockholm Convention. It even establishes disposal or recovery operations, such as physicochemical treatment, incineration on land, use as a fuel or other means to generate energy except for PCBs, and recycling/reclamation of metals and metal compounds. Pre-treatment and waste storage may take place in one of the following locations: (i) safe, deep, underground, hard rock formations; (ii) salt mines; (iii) a landfill site for hazardous wastes, provided that the waste is solidified or partly stabilized where technically feasible. Some restrictions have also been set for MP production, use, and disposal. The Directive (EU) 904/2019 [109] on the reduction of the impact of certain plastic products on the environment requires the application of measures to reduce the use of single-use plastic products such as cups for beverages, including their covers and lids, food containers for fast food or other meal ready for immediate consumption, etc., products made from oxo-degradable plastic (i.e., plastic materials that include additives which, through oxidation, lead to the fragmentation of the plastic material into micro-fragments or to chemical decomposition), and fishing gear containing plastic (in the near future). Further restrictions regard the placing on the market of the following single-use plastic products: cotton bud sticks, cutlery (forks, knives, spoons, chopsticks), plates, straws, beverage stirrers, sticks for attaching and supporting balloons, and food and/or beverage containers made of expanded polystyrene. A clearly legible and indelible marking on packaging regarding the appropriate waste management or waste disposal of sanitary towels, tampons and tampon applicators, wet wipes (i.e., pre-wetted personal care and domestic wipes), tobacco products with filters, and cups for beverages, is also required. Further responsibilities are attributed to producers regarding the costs of waste collection and the subsequent transport and treatment of single-use plastic products.

### 5.2. Wastewater Treatments

In 2019, the United Nations proclaimed the period 2021–2030 for ecosystem restoration through the cleanup of toxic wastes and any effective solution to the removal of xenobiotics [110]. Since liquid wastes spread more quickly in the environment than solid wastes, they constitute a high risk due to the possibility of reaching and polluting groundwater sources, and therefore, their management should be undertaken very carefully [111]. The initial step in wastewater treatment aims to remove suspended and floating solids through sedimentation, that is, a physical solid/liquid separation. Then, secondary treatment consists of intervention against microorganisms that consume the organic matter as food, converting it to carbon dioxide, water, and energy for their own growth. This process also removes some pollutants that settle at the bottom of the secondary settling tank, thus separating the biological sludge from the clear water. When specific substances or contaminants cannot be completely removed, water is treated individually or in combination with advanced methods like ultrasonication, ultraviolet light treatment, and exposure to ozone [112]. Some wastewater treatment technologies such as ozonation and powdered or granular activated carbon have been shown to reduce micropollutants and affect positively both the surface water and sediment polluted status [113]. Alternative treatments are based on microfiltration or ultrafiltration in a membrane bioreactor system, biofilter technology, coagulation, flocculation, magnetic extraction, chlorination associated with UV oxidation, ozonation, and activated carbon filtration [114].

Scientific evidence has demonstrated that MPs are frequently released from wastewater treatment plants and accumulate in the aquatic environment [115]. A preliminary treatment can efficiently remove the bulk of the MPs in wastewater through clarifiers or grit chambers located before the primary sedimentation tank [116]. Primary sedimentation tanks use gravity settling to remove suspended organic contaminants. The remaining plastic particles can be collected in secondary clarification tanks in which biological treatment through protozoa or metazoan utilization, or chemical treatment with ferric sulfate or polyacrylamide, may generate dissolved small particles to form microbial or chemical flocs [117].

### 5.3. Bioremediation

Additional waste treatments are based on the use of naturally occurring microorganisms that degrade hazardous pollutants as food for their development. Bioremediation is a cleaning technique based on using various bacteria, algae, fungi, and yeast to remove environmental contaminants from polluted sites [118]. Microorganisms belonging to various genera such as *Achromobacter*, *Alcaligenes*, *Xanthobacter*, *Arthrobacter*, *Pseudomonas*, *Bacillus*, *Mycobacterium*, *Corynebacterium*, *Flavobacterium*, and *Nitrosomonas* have been used to perform bioremediation [119]. Through this process, pollutants are degraded and converted to less toxic forms. Several factors (physical, chemical, and biological factors; carbon and nitrogen sources; and type of microorganisms) can affect the process of bioremediation [120]. For instance, a microbial consortium often shows higher efficiency than a single microorganism because different species work together to use all substrates in the best way possible [121]. Carbon is one of the most important nutrients that increases the metabolic activity of microbial communities, speeding up the bioremediation process to break down existing pollutants [122]. Most organic compounds such as PCBs, PAHs, and PBDEs can be degraded in the environment by microbes to obtain organic carbons and energy [123]. Regarding inorganic pollutants such as metals, microbial release of chelating agents and/or acids may increase the bioavailability of metal ions [124], which can be subjected to oxidation, methylation, reduction, precipitation, dealkylation, and other biochemical transformations [125]. PFASs do not easily disintegrate in the environment or in living systems due to their strong carbon–fluorine bonds that are resistant to biotic and abiotic degradation [126]. Some authors reported the removal mechanism of carbon-based composite photocatalysts by adsorption and photodegradation [127]. The removal of PFASs has been conducted by employing a wide variety of carbon-based materials [128], such as biochar–alginate composite beads [129], reed straw-derived biochar [130], activated spent coffee grounds biochar [131], and silver nanocomposite activated carbon [132]. The main advantages and disadvantages of the described remediation strategies are shown in Table 5.

### 5.4. Alternative Technologies and Future Perspectives

In recent years, advanced techniques that employ microorganisms and/or phytoplankton to eradicate hazardous contaminants have been increasingly used. Some bacteria have physiological mechanisms to survive in impacted environments and can be applied as biotechnological tools for bioremediation. Microbial consortia able to grow in the presence of the most common metals (Cu–Zn-Pb-Ni–Cd) in marine environments were investigated for their potential as biosorbents [133]. Marine algae such as diatoms have shown the capacity to adsorb metal ions by binding through some organic constituents of the cell walls such as peptidoglycans, polysaccharides, lipids, and proteins. The identification of the metal-binding proteins/enzymes and possible correlations with the metabolic pathways responsible for metal sequestration by diatoms has been studied by Chasapis et al., (2022) [134]. The authors reported that the proteins binding non-essential metals (Cd, Hg, Pb, Cr, As, and Ba) were significantly more than those identified for essential metals (Zn, Cu, Fe, Ca, Mg, Mn, Co, and Ni), highlighting their potential use for toxic metal reduction in the aquatic environment. Bioremediation can be further enhanced using genetic engineering techniques through the manipulation of microorganisms by introducing new genes and new plasmids into the bacterial genome, which modify the metabolic pathways and the adaptation to new environmental conditions [135]. Additional methods used to favor the microbial degradation of contaminants are bioaugmentation or biostimulation. The first involves the controlled addition of highly specialized microbial cultures to assist populations that are already present, while biostimulation is an innovative process that provides benefits through specific nutrients that speed up and make the bioremediation process more efficient [136]. Phytoremediation is an emerging green approach used to detect, degrade, and remove contaminants using plants. It generally involves the direct uptake of pollutants by plant tissue, with the elimination of volatile organic compounds through leaves via transpiration, or by releasing exudates that activate microbial activity associated with plant roots. Different aquatic plants are exploited to treat water pollution thanks to their high absorption and photosynthetic activity. *Elodea canadensis* and *Eichhornia crassipes* are macrophyte aquatic plants with fast growth. They float freely in water and are able to degrade pesticides [137]. The development of nanotechnologies producing various types of materials, including nanoparticles and nanomaterials, represents a promising innovation in the enhancement of the process of bioremediation. Nanoparticles such as Au, Cu, carbon-based nanomaterials (nanotubes), nanocomposites, and bionanomaterials are some examples of nanomaterials used [138].

## 6. Conclusions

The impact of pollution on the ecology of marine ecosystems can lead to multiple consequences that affect both the environment and marine organisms, reaching humans through the food web and causing long-term illnesses such as cancer or problems in developing children. Most environmental contaminants in fish products originate from polluted waters and potential reduction measures are strictly limited. Considering the health nutrients associated with fish consumption, a balance between risks and benefits is particularly important [139]. The environmental hazards that mostly threaten marine ecosystems, such as POPs, metals, and MPs, must be very quickly addressed. Furthermore, the combined exposure to multiple chemicals that have adverse effects on human health represents a challenge for both scientists and risk managers [140]. For contaminants of emerging interest such as MPs and NPs, for which no maximum levels are currently in force, the evolution of knowledge and scientific evidence should be a continuous process that is updated. The combination of legislation and remediation strategies could be the most appropriate approach for enhancing the responsibilities of both public and scientific communities.

## Figures and Tables

**Table 1:** Maximum levels (mg/kg) of contaminants in fish products according to Commission Regulation (EU) 915/2023 [6].

Contaminants	Fish	Cephalopods	Crustaceans	Bivalve Mollusks
Lead	0.30	0.30	0.50	1.5
Cadmium	0.050	1.0	0.50	1.0
Mercury	0.50	0.30	0.50	0.50
Sum of dioxins	3.5 × 10^−9^	3.5 × 10^−9^	3.5 × 10^−9^	3.5 × 10^−9^
Sum of dioxins and dioxin-like PCBs *	6.5 × 10^−9^	6.5 × 10^−9^	6.5 × 10^−9^	6.5 × 10^−9^
Sum of non-dioxin-like PCBs	0.000075	0.000075	0.000075	0.000075
Benzo(a)pyrene	-	-	-	0.005
Sum of PAHs **	-	-	-	0.03
Sum of PFASs ***	0.002	-	0.005	0.005

Legend: * polychlorinated biphenyls; ** polycyclic aromatic hydrocarbons; *** perfluoroalkyl substances.

**Table 2:** List of abbreviations of the investigated environmental contaminants.

Contaminants	Acronyms
Persistent organic pollutants	POPs
Polycyclic aromatic hydrocarbons	PAHs
Polychlorinated biphenyls	PCBs
Polychlorinated dibenzo-para-dioxins	PCCDs
Polychlorinated dibenzofurans	PCDFs
2,3,7,8-tetrachlorodibenzo-para-dioxin	2,3,7,8-TCDD
Polybrominated diphenyl ethers	PBDEs
Perfluoroalkyl substances	PFASs
Perfluorooctane sulfonic acid	PFOS
Perfluorooctanoic acid	PFOA
Perfluorononanoic acid	PFNA
Perfluorohexane sulfonic acid	PFHxS
Organochlorine pesticides	OCPs
Polychlorinated naphthalene	PCN
Dechlorane plus	DP
Hexachlorobenzene	HCB
Arsenic	As
Lead	Pb
Cadmium	Cd
Methylmercury	MeHg
Microplastics	MPs
Nanoplastics	NPs

**Table 3:** Monthly fish consumption limits for noncarcinogenic health endpoint based on metal concentrations (mg/kg, wet weight) associated with 12 to 15.9 meals (USEPA 2000).

Fish Meals/Month *	iArsenic **	Cadmium	Methylmercury	Selenium	Tributyltin
Unrestricted (<16)	0–0.088	0–0.088	0–0.029	0–1.5	0–0.088
16	>0.088–0.18	>0.088–0.18	>0.029–0.059	>1.5–2.9	>0.088–0.18
12	>0.18–0.23	>0.18–0.23	>0.059–0.078	>2.9–3.9	>0.18–0.23
8	>0.23–0.35	>0.23–0.35	>0.078–0.12	>3.9–5.9	>0.23–0.35
4	>0.35–0.70	>0.35–0.70	>0.12–0.23	>5.9–12	>0.35–0.70
3	>0.70–0.94	>0.70–0.94	>0.23–0.31	>12–16	>0.70–0.94
2	>0.94–1.4	>0.94–1.4	>0.31–0.47	>16–23	>0.94–1.4
1	>1.4–2.8	>1.4–2.8	>0.47–0.94	>23–47	>1.4–2.8
0.5	>2.8–5.6	>2.8–5.6	>0.94–1.9	>47–94	>2.8–5.6
None (<0.5)	>5.6	>5.6	>1.9	>94	>5.6

Legend: * the assumed meal size is 0.227 kg; ** inorganic arsenic.

**Table 4:** Monthly fish consumption limits for carcinogenic health endpoint based on PAH, PCB (mg/kg, wet weight), and dioxin/furan (pg/kg-TEQ) concentrations associated with 12 to 15.9 meals (USEPA 2000).

Fish Meals/Month *	PAHs	PCBs	Dioxins/Furans
Unrestricted (<16)	0–0.0004	0–0.0015	0–0.019
16	>0.0004–0.0008	>0.0015–0.0029	>0.019–0.038
12	>0.0008–0.0011	>0.0029–0.0039	>0.038–0.050
8	>0.0011–0.0016	>0.0039–0.0059	>0.050–0.075
4	>0.0016–0.0032	>0.0059–0.012	>0.075–0.15
3	>0.0032–0.0043	>0.012–0.016	>0.15–0.20
2	>0.0043–0.0064	>0.016–0.023	>0.20–0.30
1	>0.0064–0.013	>0.023–0.047	>0.30–0.60
0.5	>0.013–0.026	>0.047–0.094	>0.60–1.2
None (<0.5)	>0.026	>0.094	>1.2

Legend: * the assumed meal size is 0.227 kg.

**Table 5:** Comparison of remediation strategies.

Technique	Advantages	Disadvantages
Bioremediation	Cost-effective and eco-friendly	Complexity of biological materials
Physical treatments	Low-cost and easy application	Efficiency influenced by density of pollutants
Chemical treatments	Simple and available equipment	Efficiency influenced by structural characteristics of pollutants
Nanotechnology	Future applications	Low availability

## Data Availability

The original contributions presented in the study are included in the article, further inquiries can be directed to the corresponding author.
